# The Role of Qi-Stagnation Constitution and Emotion Regulation in the Association Between Childhood Maltreatment and Depression in Chinese College Students

**DOI:** 10.3389/fpsyt.2022.825198

**Published:** 2022-05-04

**Authors:** Huiyuan Huang, Quanwu Song, Jiawen Chen, Ying Zeng, Wenqi Wang, Bingqing Jiao, Jiabao Lin, Yan Li, Rong Zhang, Lijun Ma, Huafeng Pan, Yafei Shi

**Affiliations:** ^1^School of Public Health and Management, Guangzhou University of Chinese Medicine, Guangzhou, China; ^2^School of Fundamental Medical Science, Guangzhou University of Chinese Medicine, Guangzhou, China; ^3^Guangdong Provincial Hospital of Chinese Medicine, Guangdong Provincial Academy of Chinese Medical Sciences, The Second Clinical School of Guangzhou University of Chinese Medicine, Guangzhou, China; ^4^Institute of Clinical Pharmacology, Guangzhou University of Chinese Medicine, Guangzhou, China; ^5^Science and Technology Innovation Center, Guangzhou University of Chinese Medicine, Guangzhou, China

**Keywords:** childhood maltreatment, depression, Qi-stagnation constitution, emotion dysregulation, moderated mediation model

## Abstract

**Background:**

Childhood maltreatment is known as a significant risk factor for later depression. However, there remains a lack of understanding about the mechanisms through which childhood maltreatment confers risk for depression. This study explores how Qi-stagnation constitution (QSC) and emotion regulation affect the link between childhood maltreatment and depressive symptoms in Chinese college students.

**Methods:**

We recruited 2,108 college students aged 18–25 years between November 2020 and December 2021. Participants were required to complete four self-report questionnaires, including the Childhood Trauma Questionnaire-Short Form (CTQ-SF), Qi-Stagnation Constitution (QSC) subscale of the simplified Chinese Medicine Constitution Questionnaire, Difficulties in Emotion Regulation Scale (DERS), and the Beck Depression Inventory-II (BDI-II). Moderated mediation analyses were conducted.

**Results:**

There was a positive correlation between childhood maltreatment and QSC, while the QSC partially mediated the effect of childhood maltreatment on depressive scores in college students. In addition, emotion dysregulation moderated the association between QSC and depressive scores.

**Conclusion:**

These results enhance understanding of key factors influencing the link between childhood maltreatment and depressive symptoms among college students by combining the theory of TCM constitution with psychological processes. The development of strategies to prevent biased Qi-stagnation constitution and emotion dysregulation may help to improve college students’ mental health and strengthen the resilience of individuals to depression.

## Introduction

Depression is an important public health issue. The World Health Organization (WHO) predicts that depression will be the leading cause of global burden of disease by 2030 (1). Over 20 percent of Chinese college students suffered from depression and this proportion has been on the increase over the past decade (2–5). Patients with depression manifest heterogeneous symptom profiles, including persistently depressed mood, loss of interest, low self-esteem and energy level, weight loss, insomnia or hypersomnia, disturbed appetite, and disturbance in cognitive functions such as attention and memory (6). These symptoms can cause impairment to daily life and increase the risk of suicide and mortality (7). Apart from severely affecting the health of people, depression is associated with substantial financial burden (8, 9). It is essential to treat and prevent depression from various perspectives depending on the exact etiological factors due to its heterogeneous symptoms as well as the resulting difficulty in diagnosis and treatment.

There is ample evidence suggesting that childhood maltreatment is a significant risk factor for the development of depression in adulthood. Almost half of depression sufferers experienced maltreatment during their childhood (10). Childhood maltreatment, including physical and emotional neglect as well as physical, emotional and sexual abuse, is the most common psychological stressor that exerts negative effects on the healthy development among adolescents and even in adults (11). The high prevalence of childhood maltreatment has been reported both in Western countries and in China. Half of the Americans and Europeans experienced at least one childhood maltreatment (12). Meanwhile, in China, up to 64.7% of college students encountered adversity in their early life (13). Childhood maltreatment causes not only physical wounds, but also severe psychological trauma to individuals, and predisposes the sufferers to psychiatric conditions, especially for depression (14). As suggested by meta-analytic evidence (10, 15, 16), individuals subjected to prolonged and severe neglect or abuse during their childhood were put at a significantly higher risk of depression, earlier depression onset, greater disease severity and were more likely to develop chronic or treatment-resistant depression compared to those who did not experience childhood maltreatment. Although childhood maltreatment is a powerful predictor of depression, this association is not deterministic. Instead of developing depression, some individuals who have encountered severe childhood adversity demonstrate resilience (17, 18), which suggests a potentially higher complexity in the association between childhood maltreatment and depression. It is possible that childhood maltreatment is associated with depression through other variables in indirect ways. As depression remains a major public health concern among those suffering from childhood maltreatment, especially college students, research needs to focus on revealing the specific mechanism underlying the association between childhood maltreatment and later heterogeneous depression. That is, it appears necessary to gain a better understanding of the potential influencing factors or pathways between childhood maltreatment and depression, which may provide viable intervention targets for the treatment or prevention of depression.

According to Response Styles Theory (RST), depression is caused by personality and idiosyncratic tendencies of early experience (19). Similarly, Traditional Chinese medicine (TCM) believes that biased constitution is among the contributors to diseases, playing a major role in the occurrence, development, and outcome of diseases (20, 21). As a relatively stable and comprehensive natural characteristic, TCM constitution reflects a combination of various factors including physiologic function, morphology and structure, and psychological state (22, 23), which affect how you feel and behave, and how your body responds to pathogenic factors (24, 25). TCM constitution contributes to a better understanding of an individual’s overall physical and mental conditions and lays the foundation for disease prediction, prevention and treatment (26). TCM constitution can be categorized into nine types: balanced constitution, Qi-deficiency quality, Yang-deficiency quality, Yin-deficiency quality, blood stasis quality, phlegm-dampness quality, damp-heat quality, Qi-stagnation quality, and special constitution (27). Different types of constitution can predispose individuals to different disease susceptibilities and pathological processes (24, 25).

Notably, Qi-stagnation constitution (QSC) is most closely associated with depression (28). QSC is an unbalanced constitution due to long-term emotional dysfunction and stagnation of Qi movement (29). From the perspective of TCM, Qi represents the fundamental substance required for life-sustaining activities and will have an impact on individual’s mental health (30). Qi is the equivalent of “energy” or “signaling” in Western medicine. Qi stagnation is regarded as a condition in which energy metabolism or signal transduction is stagnant in the living body, which may lead to the occurrence of various diseases (31). Distinct from the neuroticism of personality psychology, Qi stagnation covers more than the category of psychology. Qi-stagnation constitution is assessed based on the shape, physiological function, psychological, and other characteristics of the human body. Qi stagnation can be manifested as pain and swelling, sentimentality, chest tightness, and taut pulse (27). Besides, the abnormality of emotional and psychological distress is also regarded as the signs of Qi stagnation. Individuals with QSC are not good at social intercourse, rather, they are quiet, introverted, fragile, sentimental, stressed, depressed, and prone to insomnia (27). A considerable number of studies have been conducted to explore the relationship between QSC and depression. For example, Liu et al. (32) revealed a positive association between QSC and depression in 1,200 female college students, and showed that the QSC could be used to significantly predict depression in women after 1 year (33). According to a review of 1,639 clinical studies (34), different types of biased constitution predisposes individuals to different disease susceptibilities. Among them, QSC accounted for the highest proportion among patients with depression. A survey of 250 patients with depression found that the proportion of patients with QSC was significantly higher than that of patients with other TCM constitutions (28). Another cross-sectional study of college students found that individuals with QSC had a significantly increased risk of major depression compared to those with balanced constitution (35). These studies are consistent in demonstrating that QSC is a risk factor for depression. Besides, TCM holds that TCM constitution is not only the result of the congenital hereditary factors, but also some acquired factors (36). The acquired factors influencing QSC development mainly include lifestyles, social and growth environment, such as unhealthy family environment and negative parenting styles (37, 38). The psychological trauma caused by the adverse environment will leave the individual emotionally distressed for a long time and gradually build up to QSC (27). Research has indicated that current life stress is associated with QSC which is a risk factor for psychological disorders such as depression (32). However, it remains unclear whether early life stress, such as childhood maltreatment is associated with QSC.

In addition, emotion regulation is suspected to be another significant factor influencing the association between childhood maltreatment and depression (39, 40). Emotion regulation refers to a variety of processes through which individuals attempt to control and manage their spontaneous flow of emotions in order to accomplish their needs and goals (41, 42). Several cross-sectional (43, 44), longitudinal (45, 46), and treatment outcome (47, 48) studies have indicated that deficits in emotion regulation contribute to the development and maintenance of depression. Other studies have provided empirical evidence that dysfunctional emotion regulation originating from childhood trauma can contribute to the development, maintenance, and treatment of depression. These studies have revealed that emotion regulation played a moderating (49) or mediating role (39, 40, 50, 51) in the association between childhood maltreatment and depression. However, evidence is inconsistent in the conclusions about the role that emotion regulation could play in the association between childhood maltreatment and depression. One possible reason for this inconsistency may be the different samples with various age ranges and gender distributions included in different studies. Another reason worth considering is that there may be other influencing factors affecting the role of emotion regulation in the pathway between childhood maltreatment and depression. Notably, among the nine types of TCM constitution, QSC is most closely related to negative emotions, emotion processing and regulation, which are known as the internal causes of psychosomatic diseases (37). TCM experts suggest that Qi stagnation often causes emotional abnormalities (52). Individuals with QSC may manifest symptoms of neurosis, showing unstable emotions and appearing depressive, thus increasing the risk of depression (53, 54). To sum up, these findings indicate the interaction effect of QSC and emotion regulation on the association between childhood maltreatment and depression, which deserves further investigation.

**FIGURE 1:**
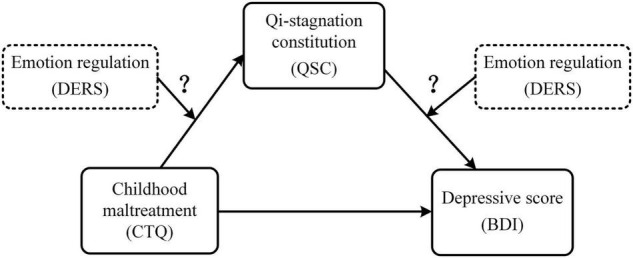
The conceptual framework of the moderated mediation model. CTQ, total score of the Childhood Trauma Questionnaire-Short Form (CTQ-SF); QSC, conversion score of the Qi-Stagnation Constitution Scale; DERS, total score of the Difficulties in Emotion Regulation Scale; BDI, depressive sore using Beck Depression Inventory-II.

In this study, we aim to explore the relationships among childhood maltreatment, QSC, emotion regulation and depression among college students by assuming a conceptual model as displayed in Figure 1. Specifically, we will first ascertain whether childhood maltreatment is associated with QSC. If the relationship holds, we will then examine whether QSC plays a mediating role between childhood maltreatment and depression. Finally, we will examine whether emotion regulation would moderate this mediation effect. From the perspective of TCM, adverse living environment and psychological barriers are the influencing factors for the formation and change of constitution and unhealthy constitution is the soil of disease. Thus, it is hypothesized that childhood maltreatment may be associated with QSC, and that QSC may mediate the effect of childhood maltreatment on the severity of depression. Furthermore, we assumed that emotion regulation would moderate the indirect association between childhood trauma and depressive scores *via* QSC.

## Materials and Methods

### Participants

We recruited 2,500 Chinese college students from Guangzhou University of Chinese Medicine to participate in this study between November 2020 and December 2021. All participants were invited to complete four self-report questionnaires (listed in the next section) in the classrooms with the guidance of well-trained investigators. Participants were excluded if they were unwilling or unable to complete the questionnaires and/or there were missing/multiple selections or obvious fictions (logical contradiction). In total, 2108 questionnaires were valid and included for analysis with an effective recovery rate of 84.32%. Informed consent was obtained from each participant before the survey. This study was approved by the local Research Ethics Committee.

### Measures

#### Childhood Trauma Questionnaire-Short Form

Severity of childhood maltreatment was assessed using the Childhood Trauma Questionnaire-Short Form (CTQ-SF), an easily administered, retrospective, self-report questionnaire (55). The 28-item CTQ-SF is a brief version of the Childhood Trauma Questionnaire and showed excellent reliability and validity (55, 56). The Chinese version of CTQ-SF adopted in the present study was translated into Chinese by Zhao et al. (56) and showed a good internal consistency. The CTQ-SF involves five sub-scales intended to assess five dimensions of childhood maltreatment (physical abuse, emotional abuse, sexual abuse, physical neglect, and emotional neglect), with each sub-scale consisting of five items. The CTQ-SF also involves three additional validity items assessing minimization/denial. The CTQ-SF uses a 5-point Likert scale to characterize the frequency of maltreatment experiences ranging from never true (score 1) to very often true (score 5). Individual was scored on each of the five items in each sub-scale with higher scores representing higher levels of being abused or neglected. The total score ranges from 25 to 125. The Cronbach’s alpha coefficient for CTQ was 0.82 in this study.

#### Qi-Stagnation Constitution Scale

Level of Qi-Stagnation Constitution (QSC) was evaluated using the Qi-stagnation Constitution Scale, a subscale of the simplified Chinese Medicine Constitution Questionnaire (57). The subscale includes four items to assess individual constitutional differences based on characteristics such as bodily sensations and psychological characteristics (e.g., Do you get anxious and worried easily? Did you feel sensitive, vulnerable, or emotionally upset? Do you have pain in your ribs or breasts? Do you have chest tightness or abdominal fullness?). Items are rated on a 5-point Likert scale ranging from “hardly any” (score = 1) to “nearly all the time” (score = 5). The total score (i.e., original score) of QSC was obtained by summing the score of all items and then convert them into one grand total (i.e., conversion score). The conversion score = (total score - lowest possible score) divided by (highest possible score - lowest possible score). The score indicates the tendency of the Qi-stagnation constitution type. This subscale has been widely used to measure the QSC in China and was found to have good validity and reliability in Chinese (58). The Cronbach’s alpha coefficient for QSC was 0.794 in this study.

#### Difficulties in Emotion Regulation Scale

The Difficulties in Emotion Regulation Scale [DERS (59)] is a 36-item self-assessment questionnaire measuring several facets of emotion regulation. The questionnaire involves difficulties relevant to an individual’s (a) emotional perception, (b) emotional understanding, (c) acceptance of emotional response, (d) control of emotional impulses, (e) difficulty in target orientation, and (f) difficulty in effective use of emotion regulation strategies. Participants rate how frequent each item applies to them on a 5-point Likert scale ranging from 1 (“almost never”; 0–10% of the time) to 5 (“almost always”; 91–100% of the time). In case of a higher score, it indicates the worse capability of emotion regulation. The Chinese version of the DERS was demonstrated to be a reliable and valid measurement (60). The Cronbach’s alpha coefficient for DERS was 0.922 in this study.

#### Beck Depression Inventory-II

Beck Depression Inventory-II (BDI-II) is a widely used self-reported scale for assessing the severity of depressive symptoms of participants during the past 2 weeks based on DSM-IV criteria (61). It involves 21 items, each with a 0–3 rating (e.g., from 0 = “I have not lost interest in other people or activities” to 3 = “It’s hard to get interested in anything”). The total score of the BDI-II ranges from 0 to 63 with higher scores indicating higher levels of depression. It has demonstrated excellent validity and reliability in samples of Chinese adults (62). The Cronbach’s alpha coefficient for BDI-II was 0.954 in this study.

### Data Analysis

The data was analyzed using SPSS 25.0 (IBM Corp., Armonk, NY, United States). Univariate descriptive statistics were performed to examine the distributions of age, childhood trauma (CTQ), Qi-stagnation constitution (QSC), difficulties in emotion regulation (DERS), and depressive symptoms (BDI) of the study participants. Pearson’s correlation analyses were conducted to examine the association between age, CTQ, QSC, DERS, and BDI. Point-biserial correlations were calculated to test the relationships between gender and the studied variables. We also calculated the Pearson’s correlation coefficient between QSC and the scores of the five subscales (physical abuse, emotional abuse, sexual abuse, physical neglect, and emotional neglect) of the CTQ-SF to further explore whether QSC was associated with different types of childhood maltreatment. Multiple linear regression analyses and a PROCESS macro program (63) were used to examine the mediating effect of QSC and the moderating effect of DERS between childhood maltreatment (CTQ) and depression scores (BDI), taking gender and age as control variables. When evaluating the moderating effect of DERS, all of the independent variables were mean-centered before the interaction terms were constructed to avoid the influence of multi-collinearity (64). Additionally, simple slope analyses were carried out to demonstrate the significant interaction at 1 Standard Deviation (SD) below the mean and 1 SD above the mean of DERS. The 95% confidence interval (CI) was calculated based on 5,000 bootstrap samplings and the significance of the point estimate (*p* < 0.05) was determined by the absence of zero within the 95% CI.

In addition, in order to examine whether gender has an effect on our model, we divided the participants into male and female groups, and repeated the data analyses above in each group, respectively, with age as a covariate.

## Results

### Demographics Characteristics and Preliminary Statistics

Among the 2,108 college students, 670 (31.8%) were males and 1,438 (68.2%) were females. The gender ratio was imbalanced but was comparable to those of previous studies of medical universities (65–68). The sample was aged 18–25 years (Mean = 18.51 years old, *SD* = 0.77). Table 1 shows the descriptive statistics of the studied variables and the percentages of different types of childhood maltreatment exposures.

**TABLE 1:** Descriptive statistics of the studied variables.

Variables	Mean	SD	Range	n/N (%)
Age (years old)	18.51	0.77	18–25	
CTQ				
Total score	34.15	9.96	25–92	
Emotional abuse	6.79	2.66	5–23	17.8%
Physical abuse	5.91	2.03	5–22	11.7%
Sexual abuse	5.44	1.53	5–21	14.3%
Emotional neglect	9.06	4.34	5–25	39.1%
Physical neglect	6.95	2.54	5–21	29.3%
Single exposure	N/A	N/A	N/A	21.9%
Multiple exposures	N/A	N/A	N/A	32.6%
QSC	27.28	19.48	0–100	
DERS	84.78	21.19	36–153	
BDI	7.26	6.82	0–47	

*N = 2108. SD, standard deviation; CTQ, Childhood Trauma Questionnaire-Short Form (CTQ-SF); QSC, conversion score of the Qi-Stagnation Constitution Scale; DERS, total score of the Difficulties in Emotion Regulation Scale; BDI, depressive sore using Beck Depression Inventory-II; n/N (%), percentage of different type of childhood maltreatment exposures.*

#### Childhood Maltreatment

The average CTQ-SF total score within our sample was 34.15 (*SD* = 9.96). A total of 1,150 participants (54.6%) reported having experienced at least one type of maltreatment during childhood. The prevalence rate was comparable to those of previous findings in the university population (13). Specifically, 17.8% endorsed having experienced some degree of emotional abuse; 11.7% endorsed having experienced some degree of physical abuse; 14.3% endorsed having experienced some degree of sexual abuse; 39.1% endorsed having experienced some degree of emotional neglect, and 29.3% endorsed having experienced some degree of physical neglect. 21.9% endorsed having experienced one type of maltreatment while 32.6% endorsed having experienced two or more types of maltreatment.

#### Symptoms of Depression

The average score on the BDI-II within our sample was 7.26 (*SD* = 6.82). 15% reported having mild to severe depressive scores. The prevalence rate was comparable to those of previous findings in Chinese university population (3–5). Specifically, 9.7% had depressive symptoms within the mild range (score of 14–19), 4.5% within the moderate range (score of 20–28), and 0.8% within the severe range (score of 29–63) based on cut-offs defined by Beck, Steer, and Brown (61).

Table 2 shows the results of bivariate correlation analyses of demographic variable (gender, age) and the four studied variables (CTQ, DERS, QSC, and BDI). The results were in line with the hypothesis that CTQ (*r* = 0.325, *p* < 0.001), QSC (*r* = 0.556, *p* < 0.001) and DERS (*r* = 0.532, *p* < 0.001) were positively correlated with depressive scores using BDI in college students. Additionally, DERS was positively correlated with both CTQ (*r* = 0.397, *p* < 0.001) and QSC (*r* = 0.619, *p* < 0.001). Moreover, there was a significant positive correlation between CTQ and QSC (*r* = 0.323, *p* < 0.001). In addition, we found a significant gender effect in QSC (*r* = 0.100, *p* < 0.01).

**TABLE 2:** Intercorrelations between the studied variables.

Variables	1	2	3	4	5
1. Gender^a^					
2. Age (years old)	0.034				
3. CTQ	0.019	0.016			
4. QSC	0.100**	0.006	0.323***		
5. DERS	0.019	0.015	0.397***	0.619***	
6. BDI	0.031	0.072	0.325***	0.556***	0.532***

*N = 2,108. Pearson’s correlation analyses were performed to investigate the association between age, CTQ, QSC, DERS, and BDI.*

*^a^Point-biserial correlations were performed to test the association between “gender” variable and other variables.*

SD, standard deviation; CTQ, total score of the Childhood Trauma Questionnaire-Short Form (CTQ-SF); QSC, conversion score of the Qi-Stagnation Constitution Scale; DERS, total score of the Difficulties in Emotion Regulation Scale; BDI, depressive sore using Beck Depression Inventory-II. **p < 0.01, ***p < 0.001.

### The Correlations Between Qi-Stagnation Constitution and Different Types of Childhood Maltreatment

We further examined whether QSC was correlated with different types of childhood maltreatment. Table 3 shows the correlation between QSC and CTQ-SF total score as well as scores of the five subscales of CTQ-SF. Bivariate correlation analyses indicated that QSC was significantly positively associated with CTQ-SF total score (*r* = 0.323, *p* < 0.001), emotional abuse (*r* = 0.352, *p* < 0.001), physical abuse (*r* = 0.215, *p* < 0.001), sexual abuse (*r* = 0.181, *p* < 0.001), emotional neglect (*r* = 0.251, *p* < 0.001), and physical neglect (*r* = 0.189, *p* < 0.001).

**TABLE 3:** The correlations between Qi-stagnation constitution (QSC) and different types of childhood maltreatment.

	CTQ	EA	PA	SA	EN	PN
Mean ± SD	34.15 ± 9.96	6.79 ± 2.66	5.91 ± 2.03	5.44 ± 1.53	9.06 ± 4.34	6.95 ± 2.54
QSC	0.323***	0.352***	0.215***	0.181***	0.251***	0.189***

The values in the second row of the table represent the Pearson’s correlation coefficients between Qi-stagnation constitution (QSC) and different types of childhood maltreatment. SD, standard deviations; CTQ, total score of the Childhood Trauma Questionnaire-Short Form (CTQ-SF); EA, emotional abuse; PA, physical abuse; SA, sexual abuse; EN, emotional neglect; PN, physical neglect; QCS, conversion score of Qi-stagnation constitution Scale. ***p < 0.001.

### The Mediation Effect of Qi-Stagnation Constitution on the Association Between Childhood Maltreatment and Depression

The results of the mediation effect of QSC on the association between CTQ and BDI was depicted in Table 4. First, Model 1 showed a positive association between CTQ and QSC (*B* = 0.323, *p* < 0.001). Second, Model 2 showed a positive association between CTQ and BDI (*B* = 0.321, *p* < 0.001). Third, after controlling for CTQ, QCS was positively associated with BDI (*B* = 0.508, *p* < 0.001) as shown in Model 3. Although CTQ was still significantly associated with depressive scores (*B* = 0.158, *p* < 0.001), its effect on depressive scores was reduced in Model 3 after controlling for QSC compared to Model 2.

**TABLE 4:** Results of moderated mediation analyses.

Predictor variable	QSC	BDI		
	Model 1	Model 2	Model 3	Model 4
Intercept	0.065	−1.323	−1.356	−1.655
**Control variables**				
Gender	−0.203***	0.050	−0.053	−0.009
Age (years old)	−0.022	0.067	0.078	0.085
**Independent variable**				
CTQ	0.323***	0.321***	0.158***	0.100***
**Mediator**				
QSC			0.508***	0.309***
**Moderator**				
DERS				0.297***
**Interaction term**				
QSC × DERS				0.166***
*R* ^2^	0.114	0.109	0.337	0.410
*F*	89.94***	85.58***	267.23***	243.22***

N = 2,108. Unstandardized regression coefficients are reported. CTQ, total score of the Childhood Trauma Questionnaire-Short Form (CTQ-SF); QCS, conversion score of the Qi-Stagnation Constitution Scale; DERS, total score of the Difficulties in Emotion Regulation Scale; BDI, depressive sore using Beck Depression Inventory-II. ***p < 0.001.

To revalidate the mediating role of QSC between CTQ and BDI, Hayes’ (63) Model 4 of SPSS Process macro was used to calculate the 95% confidence interval (CI) of the indirect effect based on 5,000 bootstrap samplings. In the calculation, gender and age were included as covariates. It was found that the indirect effects of CTQ on BDI through QSC were significant (95% CI = [0.138, 0.191]), which further confirmed that QSC mediated the association between childhood maltreatment and depressive scores in college students.

### The Moderation Effect of Emotion Regulation on the Mediation Model

**FIGURE 2:**
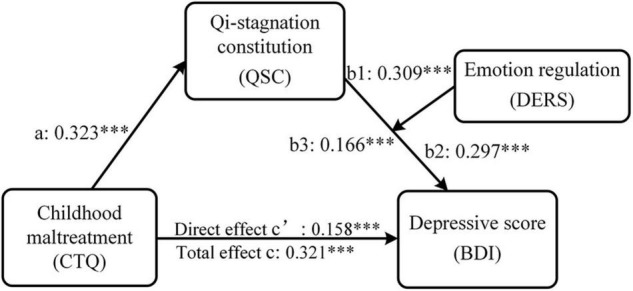
The moderated mediation model of childhood maltreatment, Qi-stagnation constitution, emotion regulation and depression. Path coefficients were shown in unstandardized regression coefficient. CTQ, total score of the Childhood Trauma Questionnaire-Short Form (CTQ-SF); QSC, conversion score of the Qi-Stagnation Constitution Scale; DERS, total score of the Difficulties in Emotion Regulation Scale; BDI, depressive sore using Beck Depression Inventory-II. ^***^p < 0.001.

**FIGURE 3:**
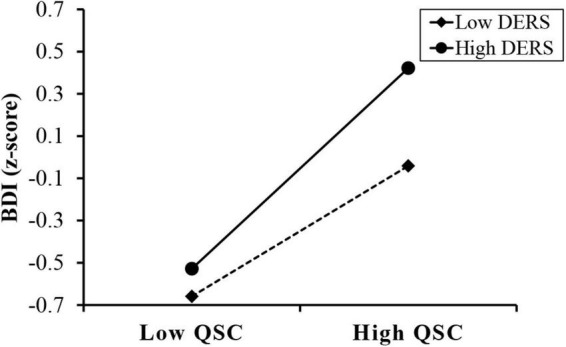
Difficulties in emotion regulation moderates the effect of Qi-Stagnation Constitution on depressive scores. QSC, conversion score of the Qi-Stagnation Constitution Scale; DERS, total score of the Difficulties in Emotion Regulation Scale; BDI, depressive sore using Beck Depression Inventory-II.

First, our results showed that the interaction between CTQ and DERS cannot predict QSC (*B* = 0.015, *p* = 0.426), indicating that DERS had no significant moderating effect on the association between CTQ and QSC. But our results showed that the interaction between QSC and DERS can predict the level of depression to a significant level (*B* = 0.166, *p* < 0.001), indicating that DERS had a moderating effect on the association between QSC and BDI as shown in Figure 2 and Table 4-Model 4. Figure 3 shows the results of the simple slope analyses used to demonstrate the significant interaction at 1 SD below the mean and 1 SD above the mean of DERS. We found that for individuals with no matter how low or high DERS, higher levels of QSC were associated with higher depressive scores (Low DERS: *B*_*simple*_ = 0.143, *t* = 4.788, *p* < 0.001; High DERS: *B*_*simple*_ = 0.475, *t* = 19.574, *p* < 0.001). Nevertheless, the relationship between QSC and depressive scores was stronger when DERS was high (1 SD above the mean) and weaker when DERS was low (1 SD below the mean).

We also use Hayes’ (63) Model 14 of SPSS Process macro to revalidate the moderated mediation effect, controlling for gender and age. The 95% confidence interval (CI) was calculated based on 5,000 bootstrap samplings. The results showed that DERS did moderate the effect of CTQ on BDI through QSC (indirect effect = 0.100, *SE* = 0.010, 95% CI = [0.081, 0.120]). The conditional indirect effects of CTQ on BDI under different levels of DERS and pairwise contrasts between conditional indirect effects were depicted in Table 5. Pairwise contrasts between conditional indirect effects of CTQ on BDI under different levels of DERS found that the indirect effect for high DERS college students (indirect effect = 0.153, *SE* = 0.014, 95% CI = [0.127, 0.183]) was significantly stronger than that of low DERS college students (indirect effect = 0.046, *SE* = 0.010, 95% CI = [0.028, 0.067]), as zero was not contained in the 95% CI presented in Table 5. That is, for individuals with higher DERS, higher childhood trauma was associated with higher depressive scores through QSC.

**TABLE 5:** Conditional indirect effects of CTQ on BDI under different levels of DERS and pairwise contrasts between conditional indirect effects.

	DERS	Effect	BootSE	BootLLCI	BootULCI
Conditional indirect effects	Effect 1 (M-1 SD)	0.046	0.010	0.028	0.067
	Effect 2 (M)	0.100	0.010	0.081	0.120
	Effect 3 (M + 1 SD)	0.153	0.014	0.127	0.183
Pairwise contrasts between conditional indirect effects	Effect 2 – effect 1	0.054	0.007	0.040	0.068
	Effect 3 – effect 1	0.107	0.014	0.080	0.137
	Effect 3 – effect 2	0.054	0.007	0.040	0.068

*CTQ, total score of the Childhood Trauma Questionnaire-Short Form (CTQ-SF); BDI, depressive sore using Beck Depression Inventory-II; DERS, total score of the Difficulties in Emotion Regulation Scale; M, mean; SD, standard deviation; BootSE, standard error of bootstrap; BootLLCI, lower limit of 95% confidence interval; BootULCI, upper limit of 95% confidence interval.*

### Testing for Gender Effects on the Moderated Mediation Model

The results on investigating the gender effects on the moderated mediation model are presented in the Supplementary Material. In brief, the same pattern of results derived from all samples was found in both male and female groups.

## Discussion

The current study investigated the potential pathway between childhood maltreatment and depressive symptoms in a sample of college students by assuming a moderated mediation model as shown in Figure 1. Consistent with our hypotheses, we first found a positive correlation between childhood maltreatment and QSC (Hypothesis 1). We then confirmed the mediating effect of QSC on the association between childhood maltreatment and depressive symptoms (Hypothesis 2), and the moderating effect of emotion regulation on the association between QSC and depressive symptoms (Hypothesis 3). These results contribute to a better understanding of the pathways by which childhood maltreatment impacts on individual depressive symptoms in college students. Implications for depression prevention and intervention are discussed.

Consistent with our hypotheses, we found a positive correlation between childhood maltreatment and QSC, while QSC partially mediated the association between childhood maltreatment and depressive score. First, consistent with prior studies (10, 15, 69–72), our results showed that childhood maltreatment was positively associated with depressive scores in college students (Table 2). This may suggest that those who experienced more neglect or abuse during childhood had a significantly increased risk of depression and more depressive symptoms in their adulthood. Furthermore, similar to previous studies (28, 32, 34, 35), we found a significantly positive correlation between QSC and depressive scores (Table 2), confirming that QSC is a predispositional and susceptible constitution of depression. Intriguingly, we found a significant gender effect on QSC as shown in Table 2. This result is consistent with the gender differences in the studied variables using independent-samples *t*-test (see Supplementary Table 2), which show that the level of QSC of female participants were significantly higher than those of male participants. These results are in line with previous epidemiological investigation of TCM constitution, which indicated that females were more likely to develop QSC compared to males (73–75). From the perspective of the psychological characteristics, females are more introverted, fragile, sentimental and possibly more vulnerable to negative emotions, thus increasing the likelihood of Qi stagnation (73). Importantly, we found consistently positive correlations between the five types of childhood maltreatment and QSC (Table 3). One possible explanation by TCM is that the liver is capable of regulating Qi movement and balancing emotions. However, the liver functions can be affected by stress (32, 76–81). For example, animal studies have revealed that responses following psychosocial challenges involve transcriptional alterations in liver tissue (76); psychological stress preferentially induces a pro-inflammatory response in the liver (81). Moreover, acute stress made an immediate impact on liver lipid metabolism for rats (77). In addition, the growing evidence obtained from human studies has suggested that psychological stress can affect liver functions (78–80). A previous review (78) suggested that psychosocial stress might influence the initiation, course and outcome of liver diseases. Fukudo et al. (79) suggested that psychosocial stress may have an impact on exacerbation of alcoholic liver injury. Early clinical reports revealed that psychosocial stress significantly decreased hepatic blood flow (80). Stress (e.g., early life stress) is known as one of the most common contributors to liver Qi stagnation in TCM (52). According to the theory of TCM constitution, TCM constitution is largely determined by the congenital endowments including ethnicity and inheritance, which contribute to the specificity and relative stability of constitution. Nevertheless, this relatively stable specificity is not necessarily static. Instead, it could be dynamically changed with the effects of acquired factors, such as social and growth environment, lifestyles, emotion, diseases, and received treatments (82–84). Prof. Qi Wang, the founder of the TCM constitution, suggested that individuals with QSC may be more sensitive to stress and more easily enter into emotional frustration when they encounter more adverse life events (27). In fact, the relationships between QSC and adverse life events in childhood have already been observed by other theorists. For example, a review by Fu et al. (82) suggested that the formation of QSC was related to the adverse experiences in childhood, such as early deprivation, parental divorce, premature death of relatives, community violence and peer bullying. Providing a further empirical support for these theories, our study suggests that level of QSC tended to be higher among the individuals reporting severer childhood maltreatment. However, all the correlations were weak in nature, although QSC was significantly correlated with various types of childhood maltreatment. The correlation coefficients between QSC and various types of childhood maltreatment ranged between 0.181 and 0.352, representing small to moderate correlation between these two variables. Given more of the variability left unexplained, there must be one or more other relevant factors linked to QSC. Therefore, clinical practitioners must interpret cautiously whether these statistically significant relationships are of clinical significance in practical terms. Future studies shall pay more attention to the factors influencing the association between childhood maltreatment and QSC. This study takes a first step to explore the potential association between childhood maltreatment and level of QSC. Given childhood maltreatment may be a risk factor for biased QSC in young adulthood, it is important to develop early intervention against childhood maltreatment to minimize the level of QSC.

More importantly, our results indicated that QSC played a mediating role between childhood maltreatment and depressive scores in college students (Table 4-Model 3). The significant mediating effect of QSC revealed that those who were subjected to severer childhood maltreatment raised their level of QSC, which, in turn, might lead to higher depressive scores. It shows that QSC acts as a risk-enhancing factor in depression among people with childhood maltreatment. Individuals with high levels of QSC show characteristics of emotional fragility and poor tolerance to mental stimulation, which may increase the vulnerability to depression (37). According to TCM theory, compared with those who do not experience an adverse life event, people who experience more adverse life events can suffer from more severe Qi stagnation in their liver, thus leading to worsened depression (32, 52). Although no research has directly shown the role of QSC in shaping the relationship between adverse childhood experiences and depression, a study by Liu et al. (32) indicated that current life stress could worsen the level of QSC thus leading to depression. As mentioned above, acquired factors may cause dynamic changes in constitution, leading to disease or health. The adjustability of constitution makes it possible to adjust and correct biased QSC, which facilitates early detection, prevention, and treatment of depression. In recent years, the theory of TCM constitution has been widely applied in clinical research and public health practices, establishing the prevention and treatment route of constitution-disease-intervention (85). TCM advocates the concept of early prevention, through reducing the level of individual QSC to prevent the development of depression. Mainstream approaches to clinical intervention for QSC include prescription, acupuncture, lifestyle modification and physical exercise, to name but a few (86). Integration of TCM constitution with western medicine is a potential alternative option toward health maintenance as well as depression prevention and alleviation (87). In general, our results may suggest childhood maltreatment as a risk factor for biased Qi-stagnation constitution, which is a potential mechanism underlying the negative effect of childhood maltreatment on depression in adulthood. This finding highlights the combined needs to strengthen family health education and carry out care interventions for maltreated individuals with high QSC to reduce risk of depression.

In addition, this study further investigated at what stage the DERS assessing emotion regulation worked regarding its moderating effect within the model. Our results revealed that DERS exerted no significant moderating effect on the association between childhood maltreatment and QSC, which may be attributed to the relatively stable TCM constitution as determined by the congenital genetic factors (27, 88). Formed in the process of individual growth and development, TCM constitution may not be easily affected by emotion regulation. However, our results indicated that the indirect effect of childhood maltreatment on depressive scores through QSC relied on the level of DERS (Table 4-Model 4). The result corresponded to previous studies showing that emotion regulation could predict depression severity (45, 89, 90) and that emotion dysregulation was an underlying factor affecting depressive symptoms in individuals with adverse childhood experiences (39, 40, 50, 51). The capability of emotion regulation is developed early in life within the context of interpersonal emotional exchanges between the caregiver and child (91). A considerable body of studies have indicated that growing up with experiences of maltreatment may adversely affect a child’s later ability to regulate emotions (92, 93). Emotion dysregulation linked to early life adversity appears to be relevant to the onset, maintenance, and treatment of depression (94). Our results extend these previous findings by showing that the association between QSC and depression symptoms relied on the level of DERS. According to the results of simple slope analyses, compared to those with lower emotion dysregulation, individuals with childhood maltreatment who tended to have QSC under higher emotion dysregulation were more likely to be scored high on depression (Figure 3 and Table 5). It seems that emotion dysregulation may serve to strengthen the disadvantages associated with QSC, thus, causing the individuals with childhood maltreatment to suffer from depression. A plausible explanation by TCM is that Qi (vital energy), which is believed to be capable of vitalizing, propelling, and warming the body, depends on the regulation of the liver, which balances emotions (52, 95, 96). If the liver malfunctions, Qi gets blocked and induces emotional abnormalities and dysregulation, thus increasing the risk of depression (52). Our results are congruent with a prior study (32) demonstrating that rumination (a maladaptive emotion regulation strategy) may affect the relationship between QSC and depression in women. Researchers (32, 97) have proposed that maladaptive emotion regulation (e.g., rumination) will make individuals focus on their own negative emotions and negative events, reduce the efficiency of solving problems, and finally cause depression. Therefore, the level of depression is not only dependent on childhood adversity and QSC, but also on emotion regulation. Effective emotion regulation or low emotion dysregulation may serve as a protective factor against depression. The effective utilization of emotion regulation function as made possible through psychological counseling, can reduce the probability of depression in individuals with adverse childhood experiences and high QSC levels. Appropriate emotion regulation skills may mitigate the effect of QSC on depression from the perspective of clinical practice.

### Limitations

Several limitations should be born in mind in this study. First, caution should be given to the interpretation of our results as the study population is restricted to general college students, which may limit the replicability of our findings to other depressive patients. The associations between childhood maltreatment, QSC, emotion regulation and depression in diverse samples such as clinically depressed patients should undergo further examinations in the future. Second, similar to previous studies (65–68), there was a gender imbalance in our sample as females outnumbered males. The skewed gender ratio toward female may be due to the fact that participants in the current study mainly came from medical universities in China, which have a gender ratio of men to women approaching 1:2. To remedy, we have controlled the effects of gender and age in our data analyses. In addition, we repeated the same analyses with the male and female groups, respectively, to examine whether gender has an effect on our model. The results showed that the same pattern of results derived from all samples was found in both male and female groups (see Supplementary Material). Third, all variables were assessed with self-report questionnaires. Retrospective assessments rely on the accuracy of the participant’s memory and may be prone to reporting bias. These could be improved in future studies through interviews with participants or using more accurate means of clinical assessment on childhood maltreatment, QSC, emotion regulation and depression instead of using self-report questionnaires. Fourth, this study only considered the overall severity of childhood maltreatment. We did not collect more detailed information about when and how long they were maltreated in childhood as well as the detailed demographic information of the participants, such as socioeconomic status (SES). Future research may benefit from collecting more comprehensive information about childhood maltreatment and demographic information to take into consideration potential confounding effects and to provide more specific conclusions about how different aspects of childhood maltreatment can contribute to depression. Fifth, this study did not consider the effects of different types of childhood maltreatment, which should be investigated in the future by recruiting more participants. Sixth, this study only examined the mediating role of QSC in the association between childhood maltreatment and depression. Since other TCM constitutions may also affect mental health, future studies should include other TCM constitutions and further analyze whether other TCM constitutions would mediate the link between childhood maltreatment and depression. Finally, the moderated mediation analyses conducted in this study are cross-sectional in nature, which limits any firm conclusions regarding causality or temporal onset of QSC, emotion dysregulation and depressive outcomes. While our results suggest the mediating role of QSC and the moderating effect of emotion regulation between childhood maltreatment and the level of depression, longitudinal prospective cohort studies and interventional studies assessing why and how childhood maltreatment can affect depression through QSC and emotion dysregulation would be informative to rule out alternative explanations of the observed effects. A longitudinal study with traumatized individuals would enable analyses of intraindividual and interindividual differences in the courses of the presumably affected variables.

## Conclusion

This study provides empirical evidence for the mediating role of QSC between childhood maltreatment and depressive scores and the moderating effect of emotion regulation on the association between QSC and depressive scores among college students. These results contribute to a better understanding of the potential factors influencing the link between childhood maltreatment and depressive symptoms from interdisciplinary perspectives, which combined the theory of TCM constitution and psychological processes. The development of preventive strategies to ameliorate biased Qi-stagnation constitution and emotion dysregulation may help with the improvement of college students’ mental health and to strengthen the resilience of individuals to depression. In consideration of the limitations of cross-sectional studies, it is essential to verify the moderated mediation model by conducting prospective cohort studies and interventional studies in the future.

## Data Availability Statement

The datasets analyzed in this article are now not publicly available because the datasets are part of an unpublished database and the database is still being used for other manuscripts in preparation. Further enquires can be directed to the corresponding author.

## Ethics Statement

The studies involving human participants were reviewed and approved by the Research Ethics Committee of South China Normal University. The patients/participants provided their written informed consent to participate in this study.

## Author Contributions

HH, HP, and YS contributed to the conception and design of the study. YZ and WW collected and analyzed the data. HH and QS drafted the manuscript. HH, QS, JC, YZ, WW, BJ, JL, LM, YL, RZ, HP, and YS provided critical comments and revisions. All authors read and approved the final version of the manuscript for submission.

## Conflict of Interest

The authors declare that the research was conducted in the absence of any commercial or financial relationships that could be construed as a potential conflict of interest.

## Publisher’s Note

All claims expressed in this article are solely those of the authors and do not necessarily represent those of their affiliated organizations, or those of the publisher, the editors and the reviewers. Any product that may be evaluated in this article, or claim that may be made by its manufacturer, is not guaranteed or endorsed by the publisher.
