# Supplementary material for: The Role of Qi-Stagnation Constitution and Emotion Regulation in the Association Between Childhood Maltreatment and Depression in Chinese College Students
Source: Front Psychiatry. 2022 May 4;13:825198. doi: 10.3389/fpsyt.2022.825198 (PMC9114459; doi:10.3389/fpsyt.2022.825198)
Supplement: Supplementary file 1 [file Data_Sheet_1.pdf]

Supplementary Materials

Results

1. Male group

1.1 Demographics characteristics and preliminary statistics

The mean (SD) age of the male sample was 18.47 (0.76) years. The means, SDs and bivariate correlations for all variables were presented in Table S1. As expected, CTQ (r = 0.341, p < 0.001), QSC (r = 0.554, p < 0.001) and DERS (r = 0.518, p < 0.001) were positively correlated with depressive scores using BDI. Additionally, DERS was positively correlated with both CTQ (r = 0.429, p < 0.001) and QSC (r = 0.618, p < 0.001). Also, there was a significant positive correlation between CTQ and QSC (r = 0.332, p < 0.001). These results were consistent with the main results derived from all samples.

Table S1 Descriptive statistics and intercorrelations between studied variables in the male and female group, respectively.

	**Variables**	**Mean**	**SD**	**Range**	**1**	**2**	**3**	**4**
	1.Age (years old)	18.47	0.76	18-25				
	2.CTQ	33.87	10.41	25-86	0.022			
**Male (n = 670)**	3.QSC	24.43	19.95	0-100	0.045	0.332***		
	4.DERS	84.20	22.10	36-145	0.017	0.429***	0.618***	
	5.BDI	6.95	6.75	0-47	0.023	0.341***	0.554***	0.518***
	1.Age (years old)	18.53	0.78	18-24				
	2.CTQ	34.28	9.75	25-92	0.028			
**Female (n = 1438)**	3.QSC	28.61	19.11	0-100	0.049	0.319***		
	4.DERS	85.06	20.75	36-153	0.018	0.380***	0.621***	
	5.BDI	7.41	6.85	0-43	0.031	0.317***	0.558***	0.539***

SD, standard deviation; CTQ, total score of the Childhood Trauma Questionnaire-Short Form (CTQ-SF); QSC, conversion score of the Qi-Stagnation Constitution Scale; DERS, total score of the Difficulties in Emotion Regulation Scale; BDI, depressive sore using Beck Depression Inventory-II. *p<0.05,**p<0.01,***p<0.001.

In addition, we also calculated the gender effects on the studied variables as shown in Table S2. The two groups of subjects did not differ with respect to age, CTQ, DERS, and BDI score. However, the two groups of subjects differed on levels of QSC (independent-samples *t*-test: t=-4.61, $p < 0.001$). The levels of QSC of female subjects (28.61±19.11) were higher than those of male subjects (24.43 ±19.95).

Table S2 Gender effects on the studied variables.

	**Male (n = 670)**		**Female (n = 1438)**		***t*-value**	***p*-value**
	Mean	SD	Mean	SD		
**Age (years old)**	18.47	0.76	18.53	0.78	-1.57	0.12
**CTQ**	33.87	10.41	34.28	9.75	-0.88	0.38
**QSC**	24.43	19.95	28.61	19.11	-4.61	＜0.001
**DERS**	84.20	22.10	85.06	20.75	-0.87	0.39
**BDI**	6.95	6.75	7.41	6.85	-1.44	0.15

SD, standard deviation; CTQ, total score of the Childhood Trauma Questionnaire-Short Form (CTQ-SF); QCS, conversion score of the Qi-Stagnation Constitution Scale; DERS, total score of the Difficulties in Emotion Regulation Scale; BDI, depressive sore using Beck Depression Inventory-II.

1.2 The correlations between QSC and different types of childhood maltreatment

Table S3 show the correlations between QSC and CTQ-SF total score as well as scores of the five subscales of CTQ-SF in the male group. Bivariate correlation analyses indicated that QSC was significantly positively associated with CTQ-SF total score ($r = 0.332$, $p < 0.001$), emotional abuse ($r = 0.378$, $p < 0.001$), physical abuse ($r = 0.226$, $p < 0.001$), sexual abuse ($r = 0.199$, $p < 0.001$), emotional neglect ($r = 0.250$, $p < 0.001$) and physical neglect ($r = 0.190$, $p < 0.001$). These results were consistent with the main results derived from all samples.

Table S3 The correlations between Qi-stagnation constitution (QSC) and different types of childhood maltreatment in the male and female group, respectively.

		**CTQ**	**EA**	**PA**	**SA**	**EN**	**PN**
**Male (n = 670)**	Mean ± SD	33.87±10.41	6.60±2.71	5.97±2.21	5.45±1.59	8.85±4.44	7.00±2.63
	QSC	0.332^{***}	0.378^{***}	0.226^{***}	0.199^{***}	0.250^{***}	0.190^{***}
**Female (n = 1438)**	Mean ± SD	34.28±9.75	6.87±2.63	5.89±1.95	5.44±1.50	9.16±4.29	6.92±2.50
	QSC	0.319^{***}	0.334^{***}	0.214^{***}	0.174^{***}	0.249^{***}	0.192^{***}

SD, standard deviations; CTQ, total score of the Childhood Trauma Questionnaire-Short Form (CTQ-SF); EA, emotional abuse; PA, physical abuse; SA, sexual abuse; EN, emotional neglect; PN, physical neglect; QCS, conversion score of Qi-stagnation constitution Scale. *p＜0.05,**p＜0.01,***p＜0.001.

1.3 Testing for the mediation effect

The results of the mediation effect of QSC on the association between CTQ and BDI in the male group was depicted in Table S4. First, Model 1 showed that there was a positive association between CTQ and QSC ($B = 0.334$, $p < 0.001$). Second, Model 2 showed a positive association between CTQ and BDI ($B = 0.342$, $p < 0.001$). Third, after controlling for CTQ, QCS was significantly positively associated with BDI ($B = 0.498$, $p < 0.001$) as shown in Model 3. Of note, though CTQ was still significantly associated with depressive scores ($B = 0.175$, $p < 0.001$), its effect on level of depression was reduced in Model 3 after controlling for QSC compared to Model 2. These results were consistent with the main results derived from all samples.

To revalidate the mediating role of QSC between CTQ and BDI in the male group, Hayes' (2013) Model 4 of SPSS Process macro was used to calculate the 95% confidence interval (CI) of the indirect effect based on 5000 bootstrap sampling. In the calculation, age was included as covariates. It was found that the indirect effects of CTQ on BDI through QSC were significant (95% CI = [0.122, 0.216]), which further confirmed that QSC mediated the association between childhood maltreatment and level of depression in the male group.

Table S4 Results of moderated mediation analyses in the male and female group, respectively.

Predictor variable	**Male (n = 670)**				**Female (n = 1438)**			
	**QSC**	**BDI**			**QSC**	**BDI**		
	Model 1	Model 2	Model 3	Model 4	Model 1	Model 2	Model 3	Model 4
Intercept	2.497	0.745	-0.498	-1.080	-0.570	-2.113	-1.824	-1.893
**Control variables**								
Age (years old)	-0.014	-0.040	0.027	0.053	0.031	0.084	0.076	0.096
**Independent variable**								
CTQ	0.334***	0.342***	0.175***	0.092**	0.317***	0.310***	0.149***	0.100***
**Mediator**								
QSC			0.498***	0.295***			0.506***	0.313***
**Moderator**								
DERS				0.289***				0.299***
**Interaction term**								
QSC×DERS				0.171***				0.163***
R^2	0.121	0.117	0.335	0.397	0.102	0.108	0.338	0.416
F	45.70***	44.28***	111.87***	87.50***	81.62***	86.89***	244.27***	203.67***

Unstandardized regression coefficients are reported. CTQ, total score of the Childhood Trauma Questionnaire-Short Form (CTQ-SF); QCS, conversion score of the Qi-Stagnation Constitution Scale; DERS, total score of the Difficulties in Emotion Regulation Scale; BDI, depressive sore using Beck Depression Inventory-II. *p<0.05,**p<0.01,***p<0.001.

1.4 Testing for the moderated mediation effect

Consistent with the main analyses, results showed that the interaction between CTQ and DERS cannot predict QSC (B= 0.052, p = 0.105), indicating that DERS had no significant moderating effect on the association between CTQ and QSC in the male group. But our results showed that the interaction between QSC and DERS can predict the level of depression to a significant level (B= 0.171, $p < 0.001$), indicating that DERS had a moderating effect on the association between QSC and BDI as shown in Figure S1 and Table S4-Model 4 of the male group. Figure S1 shows the results of simple slope analyses used to demonstrate the significant interaction at 1 SD below the mean and 1SD above the mean of DERS of the male group. We found that for males with no matter low or high DERS, higher QSC was associated with higher depressive scores (Low DERS: B_{simple} = 0.123, t = 2.119, $p < 0.05$; High DERS: B_{simple} = 0.466, t = 11.028, $p < 0.001$). But the relationship between QSC and depression is stronger when DERS are high (1SD above the mean) and weaker when DERS are low (1 SD below the mean). These results were consistent with the main results derived from all samples.

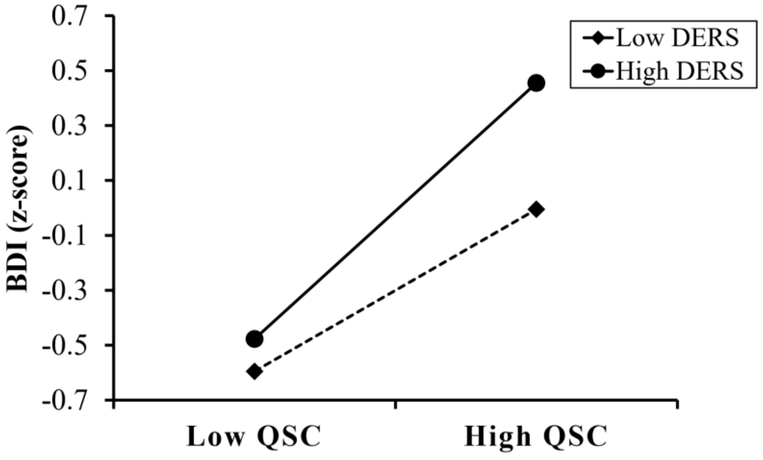

Figure S1 Difficulties in emotion regulation (DERS) moderates the effect of Qi-Stagnation Constitution (QSC) on depressive scores (BDI) in the male group.

Hayes' (2013) Model 14 of SPSS Process macro was used to revalidate the moderated mediation effect, controlling for age. The 95% confidence interval (CI) was calculated based on 5000 bootstrap

sampling. The results showed that DERS did moderated the effect of CTQ on BDI through QSC (indirect effect = 0.098, SE =0.019, 95% CI = [0.065, 0.138]) in the male group. The conditional indirect effects of CTQ on BDI under different levels of DERS and pairwise contrasts between conditional indirect effects were depicted in Table S5. Pairwise contrasts between conditional indirect effects of CTQ on BDI under different levels of DERS found that the indirect effect for high DERS males (indirect effect = 0.156, SE = 0.026, 95% CI = [0.108, 0.211]) was significantly stronger than that of low DERS college students (indirect effect = 0.041, SE = 0.022, 95% CI = [0.0002, 0.086]), as zero was not contained in the 95% CI presented in Table S5. That is, for males with higher DERS, higher childhood trauma was associated with higher levels of depression through QSC.

Table S5 Conditional indirect effects of CTQ on BDI under different levels of DERS and pairwise contrasts between conditional indirect effects in the male and female group, respectively.

		DERS	Effect	BootSE	BootLLCI	BootULCI
**Male (n = 670)**	Conditional indirect effects	effect1 (M-1SD)	0.041	0.022	0.0002	0.086
		effect2 (M)	0.098	0.019	0.065	0.138
		effect3 (M+1SD)	0.156	0.026	0.108	0.211
	Pairwise contrasts between conditional indirect effects	effect2- effect1	0.057	0.015	0.029	0.091
		effect3- effect1	0.115	0.031	0.059	0.182
		effect3- effect2	0.057	0.015	0.029	0.091
**Female (n = 1438)**	Conditional indirect effects	effect1 (M-1SD)	0.048	0.011	0.026	0.072
		effect2 (M)	0.099	0.012	0.077	0.125
		effect3 (M+1SD)	0.151	0.017	0.119	0.187
	Pairwise contrasts between conditional indirect effects	effect2- effect1	0.052	0.008	0.037	0.068
		effect3- effect1	0.103	0.016	0.074	0.137
		effect3- effect2	0.052	0.008	0.037	0.068

CTQ, total score of the Childhood Trauma Questionnaire-Short Form (CTQ-SF); BDI, depressive sore using Beck Depression Inventory-II; DERS, total score of the Difficulties in Emotion Regulation Scale; M, mean; SD, standard deviation; BootSE, standard error of bootstrap; BootLLCI, lower limit of 95% confidence interval; BootULCI, upper limit of 95% confidence interval.

2. Female group

2.1 Demographics characteristics and preliminary statistics

The mean (SD) age of the female sample was 18.53 (0.78) years. The means, SDs and bivariate correlations for all variables were presented in Table S1. As expected, CTQ (r = 0.317, p < 0.001), QSC (r = 0.558, p < 0.001) and DERS (r = 0.539, p < 0.001) were positively correlated with depressive scores using BDI. Additionally, DERS was positively correlated with both CTQ (r = 0.380, p < 0.001) and QSC (r = 0.621, p < 0.001). Also, there was a significant positive correlation between CTQ and QSC (r = 0.319, p < 0.001).

2.2 The correlations between QSC and different types of childhood maltreatment

Table S3 show the correlations between QSC and CTQ-SF total score as well as scores of the five subscales of CTQ-SF in the female group. Bivariate correlation analyses indicated that QSC was significantly positively associated with CTQ-SF total score (r = 0.319, p < 0.001), emotional abuse (r = 0.334, p < 0.001), physical abuse (r = 0.214, p < 0.001), sexual abuse (r = 0.174, p < 0.001), emotional neglect (r = 0.249, p < 0.001) and physical neglect (r = 0.192, p < 0.001).

2.3 Testing for the mediation effect

The results of the mediation effect of QSC on the association between CTQ and BDI in the female group was depicted in Table S4. First, Model 1 showed that there was a positive association between CTQ and QSC (B = 0.317, p < 0.001). Second, Model 2 showed a positive association between CTQ and BDI (B = 0.310, p < 0.001). Third, after controlling for CTQ, QCS was significantly positively associated with BDI (B = 0.506, p < 0.001) as shown in Model 3. Of note, though CTQ was still significantly associated with depressive scores (B = 0.149, p < 0.001), its effect on level of depression was reduced in Model 3 after controlling for QSC compared to Model 2. These results were consistent with the main results derived from all samples.

To revalidate the mediating role of QSC between CTQ and BDI in the female group, Hayes' (2013) Model 4 of SPSS Process macro was used to calculate the 95% confidence interval (CI) of the indirect effect based on 5000 bootstrap sampling. In the calculation, age was included as covariates.

It was found that the indirect effects of CTQ on BDI through QSC were significant (95% CI = [0.130, 0.194]), which further confirmed that QSC mediated the association between childhood maltreatment and level of depression in the female group.

2.4 Testing for the moderated mediation effect

Consistent with the main results derived from all samples, results showed that the interaction between CTQ and DERS cannot predict QSC (B= 0.008, p = 0.740), indicating that DERS had no significant moderating effect on the association between CTQ and QSC in the female group. But our results showed that the interaction between QSC and DERS can predict the level of depression to a significant level (B= 0.163, p < 0.001), indicating that DERS had a moderating effect on the association between QSC and BDI as shown in Figure S2 and Table S4-Model 4 of the female group. Figure S2 shows the results of simple slope analyses used to demonstrate the significant interaction at 1 SD below the mean and 1SD above the mean of DERS of the female group. We found that for females with no matter low or high DERS, higher QSC was associated with higher depressive scores (Low DERS: B_{simple} = 0.150, t = 4.358, p < 0.001; High DERS: B_{simple} = 0.477, t = 16.075, p < 0.001). But the relationship between QSC and depression is stronger when DERS are high (1SD above the mean) and weaker when DERS are low (1 SD below the mean). These results were consistent with the main results derived from all samples.

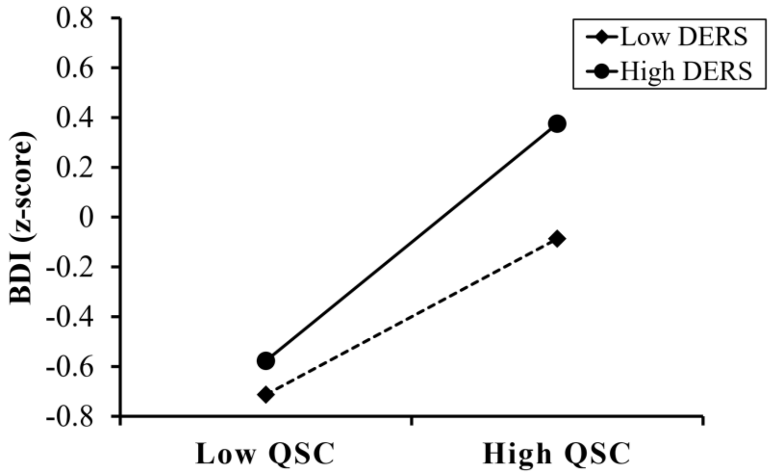

Figure S2 Difficulties in emotion regulation (DERS) moderates the effect of Qi-Stagnation Constitution (QSC) on depressive scores (BDI) in the female group.

Hayes' (2013) Model 14 of SPSS Process macro was used to revalidate the moderated mediation effect, controlling for age. The 95% confidence interval (CI) was calculated based on 5000 bootstrap sampling. The results showed that DERS did moderated the effect of CTQ on BDI through QSC (indirect effect = 0.099, SE =0.012, 95% CI = [0.077, 0.125]) in the female group. The conditional indirect effects of CTQ on BDI under different levels of DERS and pairwise contrasts between conditional indirect effects were depicted in Table S5. Pairwise contrasts between conditional indirect effects of CTQ on BDI under different levels of DERS found that the indirect effect for high DERS males (indirect effect = 0.151, SE = 0.017, 95% CI = [0.119, 0.187]) was significantly stronger than that of low DERS college students (indirect effect = 0.048, SE = 0.011, 95% CI = [0.026, 0.072]), as zero was not contained in the 95% CI presented in Table S5. That is, for females with higher DERS, higher childhood trauma was associated with higher levels of depression through QSC.

3 Associated risk factors with depressive symptoms: univariate analysis

Characteristics between students with depressive symptoms and without depressive symptoms were compared using independent sample *t*-tests and Chi-square tests. Cohen's d values were calculated to estimate effect sizes of group differences in continuous variables. Cramer's V values were calculated to evaluate the correlation between two categorical variables. Table S6 summarizes demographic (age, gender), different types of childhood maltreatment, number of types of childhood maltreatment exposures, Qi-stagnation constitution and emotion regulation by depressive symptoms status. We found that individuals with higher levels of childhood maltreatment (no matter what type of maltreatment), more types of childhood maltreatment exposures, higher levels of QSC and higher levels of DERS were associated with increased risk of depressive symptoms.

Table S6 Demographics, childhood maltreatment, Qi-stagnation constitution and emotion regulation by depression status

	**Non-depressive symptom(n=1793)**		**Depressive symptom(n=315)**		***t*/$\chi 2$**	***p*-value**	**Cohen's d/ Gramer's V**
	Mean	SD	Mean	SD			
**Age (years old)** [a]	18.49	0.75	18.60	0.89	-2.13	0.034	-0.13
**Gender, n(%)** [b]					0.29	0.319	0.01
Male	574 (32%)		96 (30.5%)				
Female	1219 (68%)		219 (69.5%)				
**CTQ score** [a]							
Total score	33.10	9.21	40.17	11.82	-10.11	＜0.001	-0.67
Emotional abuse	6.52	2.40	8.31	3.43	-8.93	＜0.001	-0.60
Physical abuse	5.80	1.88	6.58	2.66	-4.99	＜0.001	-0.34
Sexual abuse	5.36	1.33	5.92	2.30	-4.23	＜0.001	-0.30
Emotional neglect	8.63	4.14	11.52	4.65	-10.31	＜0.001	-0.66
Physical neglect	6.79	2.41	7.84	3.03	-5.86	＜0.001	-0.38
**CT exposures, n(%)** [b]					109.83	＜0.001	0.23
No exposure	889 (49.6%)		69 (21.9%)				
Single exposure	394 (22%)		68 (21.6%)				
Multiple exposures	510 (28.4%)		178 (56.5%)				
**QSC** [a]	23.96	17.72	46.21	18.17	-20.48	＜0.001	-1.24
**DERS** [a]	81.25	19.79	104.90	17.28	-21.90	＜0.001	-1.27

[a] independent sample *t*-tests; [b] Chi-square tests; SD, standard deviation; CTQ, total score of the Childhood Trauma Questionnaire-Short Form (CTQ-SF); QCS, conversion score of the Qi-Stagnation Constitution Scale; DERS, total score of the Difficulties in Emotion Regulation Scale; BDI, depressive sore using Beck Depression Inventory-II.
